# Optimizing the Performance of the Visual P300-Speller Through Active Mental Tasks Based on Color Distinction and Modulation of Task Difficulty

**DOI:** 10.3389/fnhum.2019.00130

**Published:** 2019-04-17

**Authors:** Qi Li, Zhaohua Lu, Ning Gao, Jingjing Yang

**Affiliations:** School of Computer Science and Technology, Changchun University of Science and Technology, Changchun, China

**Keywords:** brain-computer interface, visual P300-speller, active mental task, color distinction, task difficulty

## Abstract

**Objective**: P300-speller is the most commonly used brain-computer interface (BCI) for providing a means of communication to patients with amyotrophic lateral sclerosis. However, the performance of the P300-speller BCI is still inadequate. We investigated whether the performance of P300-speller can be further improved by increasing the mental effort required of the user.

**Methods**: We designed two active mental tasks for a P300-speller based on a differently colored smiling cartoon-face paradigm. The tasks were based on color distinction, and their difficulty was modulated. One of the active mental tasks (DC task) required participants to focus on and distinguish the color of a target, while the other task (CN + DC task) required participants to simultaneously count the number of times a target flashed and distinguish its color.

**Results**: The amplitudes of the event-related potentials (ERPs) in both DC and CN + DC tasks were higher than that in the CN task. The significant difference in the amplitudes between the DC and CN tasks was observed around the parietal-central area from 440 to 800 ms (late positive component, LPC), and that between the CN + DC and CN tasks was observed around the left-frontal and right-frontal areas from 320 to 480 ms (P3a) and the parietal-central area from 480 to 800 ms (P3b and LPC). The latency of the P300 potential in the CN + DC task was significantly longer than that in the CN task at F3, Fz, F4, C4, Pz, and P4 (*P* < 0.05). Offline (*P* < 0.05 at superposing once, twice, and thrice) and online (*P* < 0.001) classification results showed that the average accuracies in the CN + DC task were significantly greater than that in the CN task. Similar results were found for online information transfer rates (ITRs; *P* < 0.001). In addition, we found that the average online accuracies in the DC task were greater than those in the CN task, although the difference was not statistically significant (*P* = 0.051).

**Significance**: The active mental task based on task difficulty modulation can significantly improve the performance of the P300-speller, and that based on color distinction shows a trend of improved performance.

## Introduction

Brain-computer interface (BCI) provides a direct channel of communication between the human brain and external devices (Wolpaw et al., 2002; Wolpaw, 2004; McFarland and Wolpaw, 2011). It translates the intent or “mind” of the users into computer commands using electroencephalogram (EEG) signals from the scalp. This technology can help patients with amyotrophic lateral sclerosis communicate with the external world (Kübler et al., 2001; Nijboer et al., 2008; Mak et al., 2012; McCane et al., 2015).

P300 is an event-related potential (ERP) induced by an oddball event (Allison and Pineda, 2003). Farwell and Donchin (1988) first applied the P300 potential to the P300-speller, which is a classic BCI application for typing characters (Farwell and Donchin, 1988). Thirty-six characters arranged in a 6 × 6 matrix are presented on a display in the P300-speller. The six rows and six columns of the matrix are intensified in a random series. When a user wants to output a character, he/she need only focus on the target character. The probability of the target character’s intensity is 1/6 and is, therefore, an oddball event that will elicit the P300 potential. The system can output a character by analyzing the P300 potentials that were elicited by the intensified row and column containing the target character. Through the P300-speller, users can spell phrases or sentences by focusing on target characters. However, the performance of the P300-speller is not yet satisfactory due to its low speed and accuracy (Farwell and Donchin, 1988; Kaufmann et al., 2011).

A considerable amount of research has been conducted to design different paradigms for the improvement of the performance of the P300-speller (Pires et al., 2012; Li et al., 2013; Xu M. et al., 2013; Yin et al., 2013, 2014; Wang et al., 2015). Utilizing face stimuli to induce more distinct potentials or potentials with more components have especially helped improve the performance of the P300-speller. The characters of the P300-speller were overlaid with translucent images of famous faces (e.g., Albert Einstein, a great scientist or Yaoming, a famous basketball player) or smiling cartoon faces instead of flashing, and it was found that the face stimuli can elicit N170, N400, and vertex positive potentials in addition to the P300 potential, and these ERPs significantly improved the performance of the P300-speller (Kaufmann et al., 2011; Zhang et al., 2012; Chen et al., 2015). The color of the stimuli is one of the main factors influencing the P300 potential. Different amplitudes of P300 potentials are induced when people focus on objects with different colors; this phenomenon is attributed to the degree of color awakening (Cuthbert et al., 2000). It has, therefore, been proposed that the performance of the P300-speller can be further improved by using stimuli of different colors (Takano et al., 2009; Treder et al., 2011; Li et al., 2015; Chen et al., 2016). For instance, Li et al. (2015) designed a P300-speller by combining the use of green and familiar faces, which evoked four more obvious ERPs (P300, vertex positive potential, N170, and P600f) and improved both accuracy and information transfer rate (ITR). Guo et al. (2008) used colored vertical lines, which induced a higher-amplitude N200 in the multi-focal visual evoked potential-based BCI spelling paradigm, and thus, improved the implementation of the spelling system (Hong et al., 2009; Liu et al., 2010).

The subjects’ task in a typical P300-speller is to count the number of intensified target characters. Recent studies have improved the performance of an auditory P300-speller by designing different active mental tasks for the subjects (Guo et al., 2010; Xu H. et al., 2013). For example, Guo et al. (2010) changed the subjects’ task in an auditory P300-spelling paradigm by requiring them to simultaneously focus on the target and distinguish the direction of the sound or the sex to which it belongs. The paradigm induced a significant N200 and late positive component (LPC), and achieved better performance (Xu H. et al., 2013). The P300 potentials induced by visual stimuli usually feature larger amplitudes and longer latencies than those induced by auditory stimuli (Romero and Polich, 1996; Comerchero and Polich, 1999). Therefore, we hypothesized that the visual P300-speller can be further optimized using an active mental task.

Compared with a simple task, increasing the difficulty of discriminating the target (i.e., increasing the difficulty of the task) can induce more obvious ERPs. In the auditory BCI based on active mental tasks, Xu H. et al. (2013) designed different tasks for the subjects, increasing the difficulty without changing the attributes of the stimuli; this design induced more obvious ERPs than a simple task. Horat et al. (2016) used an auditory oddball paradigm to record the change in the ERP (P2, P3a, and P3b) amplitudes when performing the active mental task at different levels of difficulty and found that more difficult mental tasks induced more obvious amplitudes.

Based on a P300-speller with a colored smiling cartoon face paradigm, in the present study, we conducted two experiments with different active mental tasks: (1) distinguishing the target’s color; and (2) simultaneously counting the number of intensified targets and distinguishing their respective colors (a more difficult task). We compared the performance on these visual P300-spellers with that on a traditional task to explore the influence of active mental tasks (distinguishing the targets’ color) and the modulation of their difficulties on the performance of the P300-speller.

## Materials and Methods

### Subjects

Twenty-seven subjects (13 men, aged 21–27 years) were recruited to the study. Eleven subjects each participated in Experiments 1 and 2. Ten subjects (five of whom had participated in Experiment 1 or 2) participated in the online experiment. The subjects did not have any known neurological disorders and had normal or corrected-to-normal vision. No subject had color blindness. All subjects were native Chinese speakers and were familiar with the alphabet used in this study. After receiving a full explanation of the purpose and risks of the study, subjects provided written informed consent. The study was approved by the ethics committee of Changchun University of Science and Technology. All methods were performed in accordance with the approved guidelines. Before the experiment, the subjects were required to have a healthy mental state and sufficient energy to complete the experimental tasks. The whole process was conducted under the premise of volition of the subjects, and when they were in a relaxed state. All participants were compensated with 100 RMB after the completion of the experiments.

### Experimental Paradigm and Tasks

#### Experimental Paradigm

**Figure 1:**
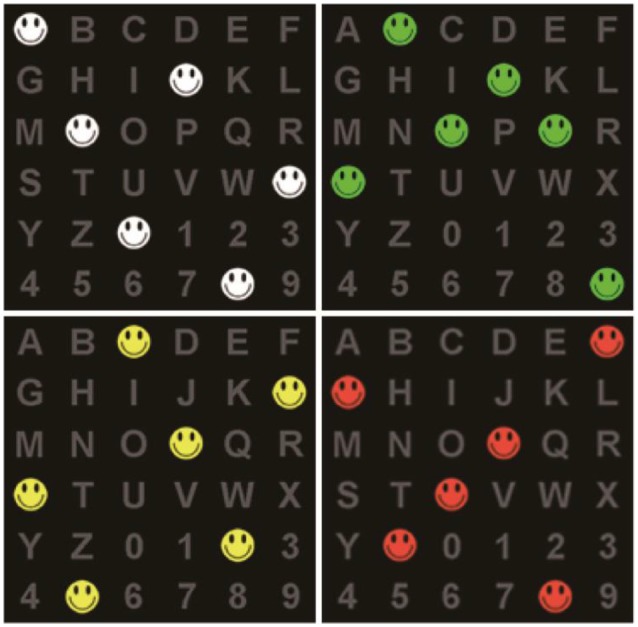
The spelling matrix with colored smiling cartoon faces.

We designed a spelling paradigm utilizing smiling cartoon faces similar to those used by Chen et al. (2015). Thirty-six characters were arranged in a 6 × 6 matrix, presented at a size of 30 cm × 30 cm on a 21.8-inch screen with a refresh rate of 60 Hz (Figure 1). The size of each character was 2.5 cm × 2.5 cm. The distance between two characters was 5 cm. The background color was black, and the characters were gray.

**Figure 2:**
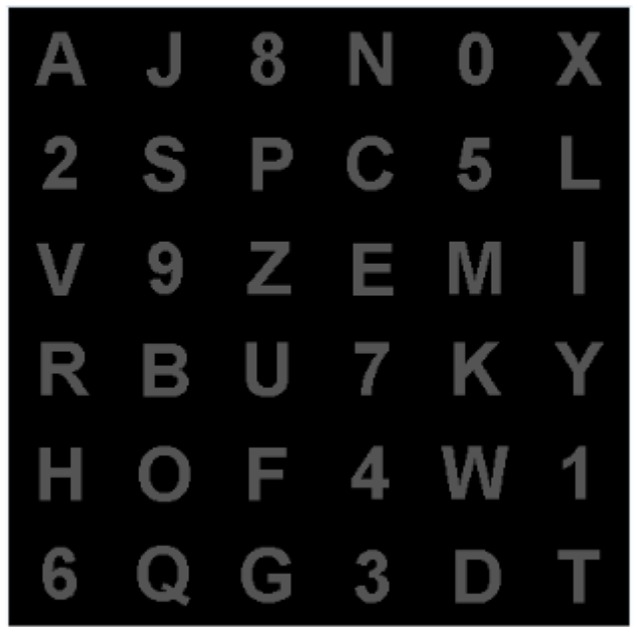
The logical matrix of the experimental paradigm.

To mitigate the problem of adjacency flashing, we used rows and columns of the logic matrix when characters were intensified, involving random rearrangement of the characters into a new matrix, as shown in Figure 2 (Townsend et al., 2010). The rows and columns were composed of six random characters in the actual matrix (Figure 1).

Flashing was performed by covering six random characters with differently colored smiling faces: red, yellow, green, or white.

#### Experimental Tasks

##### DC Task—Distinguishing a Target’s Color

In the DC task, we required subjects to conduct an active mental task: distinguishing the color of the cartoon face. A smiling face covered the target character, and the subjects said the color’s name when the target character flashed.

##### CN + DC Task—Counting the Number of Intensified Targets and Distinguishing the Targets’ Colors Simultaneously

In the CN + DC task, the subjects were not only asked to count the number of smiling faces covering the target characters when they flashed but also to distinguish their colors. For example, if the target character was “A,” four smiling faces of different colors would be presented once or twice on “A,” respectively: red, yellow, yellow, green, green, and white. The subjects were needed to silently associate the order in which the colors appeared: 1, red; 2, yellow; 3, yellow; 4, green; 5, green; 6, white.

As a control, we employed the same task as that used in the traditional P300-speller: we asked the participants to count the number of intensified targets (CN task) and ignore their colors. In the present study, we verified the performances in the DC and CN + DC tasks by comparing their offline and online results with those of the control task (CN task).

### Experimental Procedures

#### Experiment Setup, Offline and Online Protocols

The present study included two offline experiments (Experiments 1 and 2) and one online experiment. Each subject sat comfortably in front of the monitor. Their eyes were approximately 70 cm from the computer monitor. The subjects were familiar with the experimental tasks prior to commencement.

**Figure 3:**
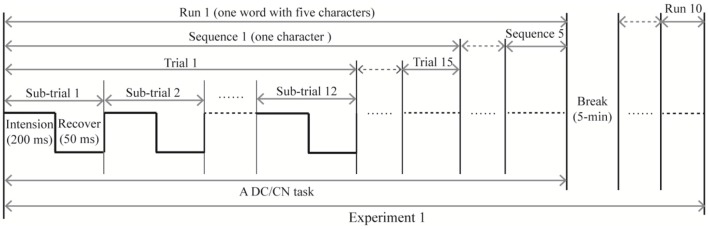
The time course of the offline experiment. The time course of Experiment 2 is similar to that of Experiment 1, with the DC task being replaced with the CN + DC task. CN: task involving counting the number of intensified targets, DC: task involving distinguishing the targets’ color.

In Experiment 1, subjects participated in two separate sessions that respectively presented the DC and CN tasks. Similarly, subjects in Experiment 2 participated in two separate sessions that presented either the CN + DC or CN task. In the offline experiments, the stimulus onset asynchrony of the flashing row/column was set at 250 ms: each character changed to the cartoon smiling face for 200 ms and then reverted to a gray character for 50 ms (Figure 3). One flash of a row or column of the logic matrix was referred to as a sub-trial. Twelve flashes corresponding to the six rows and six columns were defined as a trial. A sequence included 15 trials; thus, each sequence consisted of 180 flashes to output a target character. The output of a word with five characters was defined as a run. Between each run, subjects were permitted a 5-min break. Each subject took part in five runs (five words) for each task in Experiments 1 and 2. In each task, the five words were presented in a pseudorandom order, and the order of the two tasks was also pseudorandomized to avoid learning effects.

The online experiment was implemented on a different day. Subjects were tested with three tasks (CN, DC, and CN + DC tasks), each composed of two phases: training and testing. In the two phases, the sub-trial and trial settings were similar to those used in the offline experiment; however, trials were only repeated thrice in each sequence. There were 80 characters for each task in the training phase (output of 20 characters per training session, four training sessions), which were inputted into the classifier to learn. The test phase outputted a total of 30 characters. Experimental procedures (including offline and online procedures) were performed following the standard biosecurity and institutional safety procedures.

#### Data Acquisition

**Figure 4:**
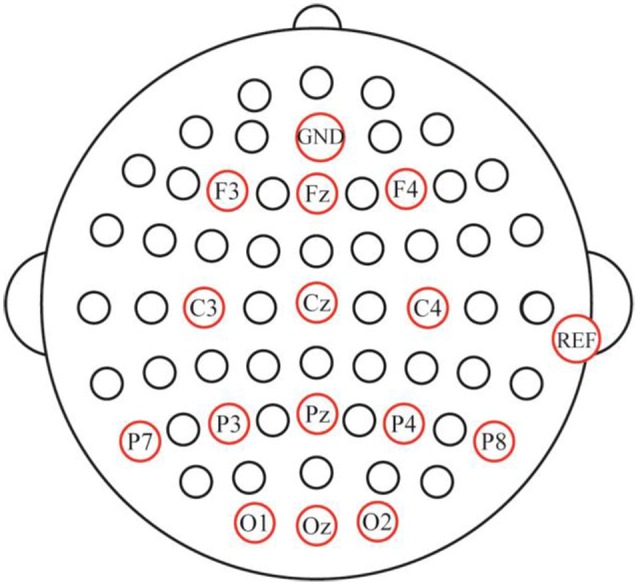
Electrode setup and locations.

In the offline experiments, electroencephalogram (EEG) data from 14 channels (Fz, F3, F4, Cz, C3, C4, Pz, P3, P4, P7, P8, Oz, O1, and O2; Figure 4) were recorded by means of an EEG amplifier (SynAmps 2; Neuroscan Inc., Abbotsford, VIC, Australia) with the AFz channel used as the ground. Vertical and horizontal eye movements were measured by the VEO and HEO electrodes, respectively, and the reference electrode was placed on the mastoid of the right ear. The impedance was maintained below 5 KΩ. All signals were digitized at a rate of 250 Hz. The EEG data were digitally filtered with a band-pass filter of 0.01–100 Hz. The stimulus presentation was controlled using a personal computer running E-prime 2.0 software (PST Inc., Savannah, GA, USA). The EEG data was acquired using NeuroScan 4.5 software (NeuroScan Inc., Phoenix, AZ, USA). In the online experiment, the ground and reference electrodes were set up in the same way as in the offline experiments. The presentation of stimuli was controlled using the MATLAB software (MathWorks Inc., Natick, MA, USA).

#### EEG Processing and Feature Extraction

For offline data, the original EEG data were first corrected for ocular artifacts with VEOG using a regression analysis algorithm and were then digitally filtered using a band-pass filter of 0.01–30 Hz. The EEG signals were divided into epochs from 100 ms before the onset of each trial to 800 ms after the onset. Baseline corrections were made against −100 to 0 ms. We then used bior4.4 tool in MATLAB to decompose the EEG signals in three dimensions and reconstructed the data using a low-frequency coefficient. We used decomposed and reconstructed data as a feature. The EEG data was down-sampled from 250 to 62.5 Hz by selecting every fourth sample from the epoch (33 time points/epoch). Because we used 14 channels, the size of the feature vector was 14 × 33. The mean amplitudes were calculated for all the electrodes at a consecutive 20-ms window between the stimulus onset and 800 ms after stimulus presentation. The data were then analyzed using a one-way repeated measure analysis of variance with the within-subjects factors of tasks (CN and DC in Experiment 1, CN and CN + DC in Experiment 2), time windows (40 levels), and electrodes (14 levels). In order to determine the electrodes and time periods in which there was a significant difference between the CN and DC/CN + DC tasks, a multiple comparison was conducted with the within-subject factors of two tasks (CN and DC/CN + DC) × 40 time windows × 14 electrodes. All statistical analyses were conducted using the SPSS version 19.0 software package (IBM Corp., Armonk, NY, USA).

For online EEG data, a third-order Butterworth bandpass filter between 0.1 and 30 Hz was used. The EEG data was then down-sampled from 250 to 62.5 Hz by selecting every fourth sample from the filtered data. We extracted the data between 150 and 750 ms after stimulus presentation (this epoch included the specific waveforms induced by the oddball event and the face stimuli) in order to reduce the computation time.

#### Classification Scheme

Bayesian linear discriminant analysis was used to classify the EEG data in the offline and online experiments; this is an extension of Fisher’s linear discriminant analysis, which avoids overfitting. The details of the algorithm can be found in previous studies (Hoffmann et al., 2008; Jin et al., 2014a,b). We used five-fold cross-validation to calculate the individual accuracies in the offline experiment: we sequentially selected one of the five words as the test, and obtained five different training and test groups; the accuracy of each of the five groups was computed, and the accuracy of each subject was obtained by averaging the five results. In the online experiment, data acquired from the training phase were used to train the classifier using Bayesian linear discriminant analysis and obtain the classifier model. The model was then used in the test phase. If there was a tie between multiple characters, the classifier would automatically select the last output as the target character.

#### Information Transfer Rate

ITR is generally used to evaluate the communication performance of a BCI system and is a standard measure that accounts for accuracy, the number of possible selections, and the time required to make each selection (Wolpaw et al., 2002). The ITR (bits min-1) can be calculated as follows:

$$\text{ITR}=\frac{60(P\log_{2}(P)+(1-P)\log_{2}\frac{1-P}{N-1}+\log_{2}N}{T}$$

where *P* denotes the probability of recognizing a character, *T* denotes the time taken to recognize a character, and *N* denotes the number of classes (*N* = 36).

## Results

### ERP Results

**Figure 5:**
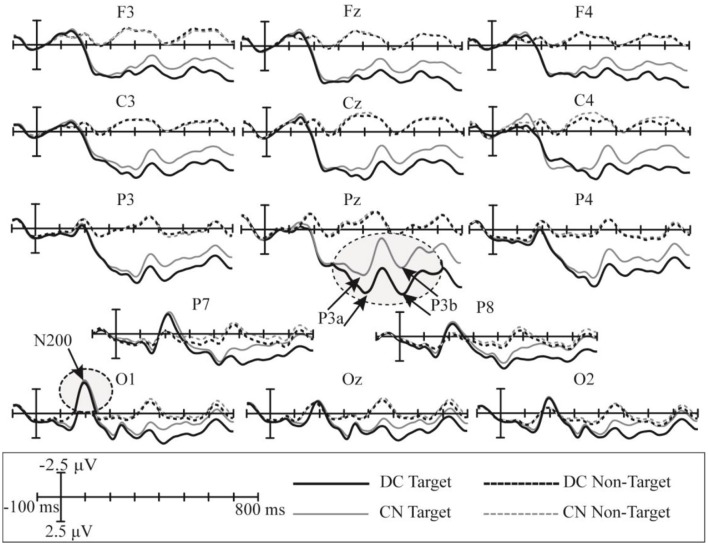
Superimposed grand-averaged waveforms of the event-related potentials (ERPs) elicited by non-target and target stimuli in the CN and DC tasks of Experiment 1. CN: task involving counting the number of intensified targets, DC: task involving distinguishing targets’ color.

The superimposed grand-averaged ERP waveform elicited by non-target and target stimuli in the CN and DC tasks are shown in Figure 5; this figure shows that the target stimuli induce larger amplitudes than the non-target stimuli at all 14 electrodes in both DC and CN tasks. A clear positive peak was clearly observed at P3, Pz, and P4 between 200 and 600 ms, which indicates the P300 potential. In addition, a clear negative waveform was observed between 100 and 300 ms at the P7, P8, O1, Oz, and O2 electrodes, which is the N200 potential. Therefore, the P300 and N200 potentials were successfully induced in the DC and CN tasks of Experiment 1.

**Figure 6:**
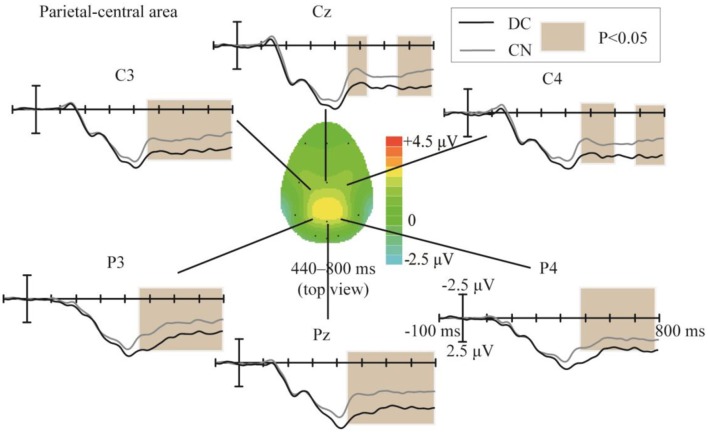
Comparison of the waveforms obtained during the DC and CN tasks, and scalp topographies at the parietal-central area between 440 and 800 ms obtained from the difference in the waveforms generated by subtracting the waveforms of the ERPs elicited during CN task from that elicited during the DC task. The color-bar represents the magnitude of the activation of the scalp areas. CN: task involving counting the number of intensified targets, DC: task involving distinguishing targets’ color.

The topographic regions corresponding to the statistically significant difference between the waveforms elicited in the DC and CN tasks are shown in Figure 6. The statistically significant difference between the ERP waveform elicited in the DC and CN tasks was found at the parietal-central area from 440 to 800 ms (*F*_(1,10)_ = 16.46, *P* < 0.05).

**Figure 7:**
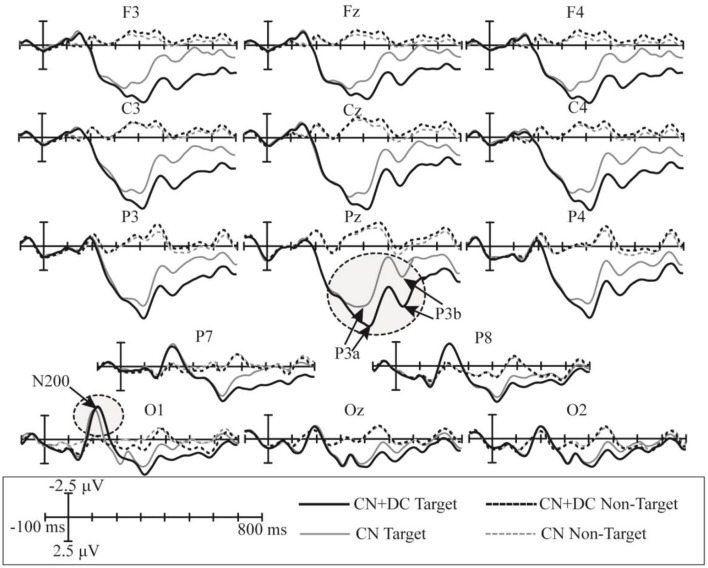
Superimposed grand-averaged waveforms of the ERPs elicited by non-target and target trials in the CN and CN + DC tasks of Experiment 2. CN: task involving counting the number of intensified targets, CN + DC: task involving simultaneously counting the number of intensified targets and distinguishing their color.

The superimposed grand-averaged ERP waveforms elicited by non-target and target trials in the CN and DC + CN tasks are shown in Figure 7. Similarly, the target stimuli induced larger amplitude waveforms than the non-target stimuli at all 14 electrodes. In both tasks, a P300 potential was observed between 200 and 600 ms in the P3, Pz, P4, P7, and P8 electrodes for the target trials. In addition, there was a N200 potential between 100 and 300 ms in the P7, P8, O1, Oz, and O2 electrodes. Therefore, P300 and N200 potentials were successfully induced in the CN + DC and CN tasks of Experiment 2.

**Figure 8:**
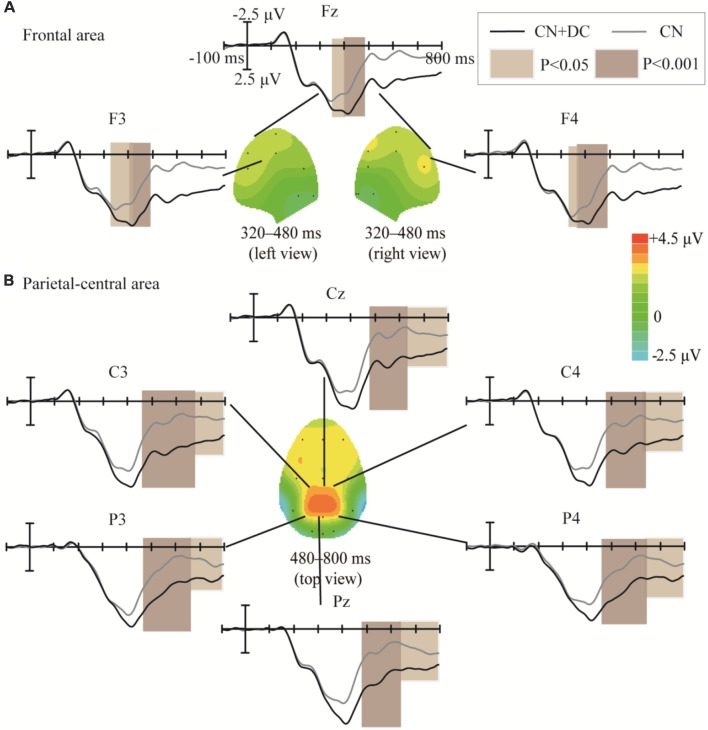
Comparison of the waveforms of the ERPs elicited during the CN + DC and CN tasks, and scalp topographies obtained from the difference in the waveforms generated by subtracting the waveforms elicited in the CN + DC task from that elicited in the CN task. **(A)** Results for the frontal area at 320–480 ms. **(B)** Results for the parietal-central area at 480–800 ms. The color-bar represents the magnitude of the activation of the scalp areas. CN: task involving counting the number of intensified targets, CN + DC: task involving simultaneously counting the number of intensified targets and distinguishing their color.

The topographic regions corresponding to the statistically significant differences between the waveforms elicited by the CN + DC and CN tasks are shown in Figure 8. The statistically significant difference between the ERP waveform elicited in the CN + DC and CN tasks was found at the left- and right-frontal areas from 320 to 480 ms (*F*_(1,10)_ = 14.54, *P* < 0.005; Figure 8A), and around the parietal-central area from 480 to 800 ms (*F*_(1,10)_ = 22.56, *P* < 0.02; Figure 8B), especially between 480 and 620 ms (*F*_(1,10)_ = 51.89, *P* < 0.001; Figure 8B).

**Figure 9:**
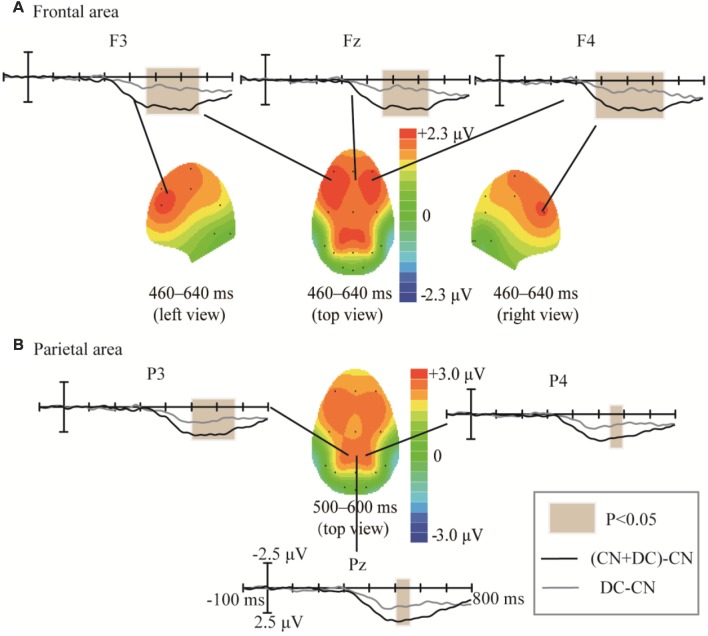
Comparison of waveforms of the ERPs for (CN + DC)-CN and DC-CN, and the corresponding scalp topographies. **(A)** Difference in the frontal area was found at 460–640 ms. **(B)** Difference in the parietal area was found at 500–600 ms. The color-bar represents the magnitude of activation of the scalp areas. CN: task involving counting the number of intensified targets, DC: task involving distinguishing targets’ color, CN + DC: task involving simultaneously counting the number of intensified targets and distinguishing their color (CN + DC)-CN: the waveform generated by subtracting waveform in CN task from that in CN + DC task. DC-CN: the waveform generated by subtracting waveform in CN task from that in DC task.

We subtracted the waveform elicited in the CN task from that elicited in the DC task, as well as the waveform elicited in the CN task from that elicited in the CN + DC task; we compared these two results in order to assess the effects of task difficulty (Figure 9). The amplitude of the difference between the CN + DC and CN waveforms in Experiment 2 was larger than the amplitude of the difference between the DC and CN waveforms in Experiment 1. These statistically significant differences in the ERP waveforms were found around the left- and right-frontal areas from 460 to 640 ms (*F*_(1,10)_ = 18.209, *P* < 0.02; Figure 9A), and the parietal area from 500 to 600 ms (*F*_(1,10)_ = 10.401, *P* < 0.05; Figure 8B).

### Latency

**Figure 10:**
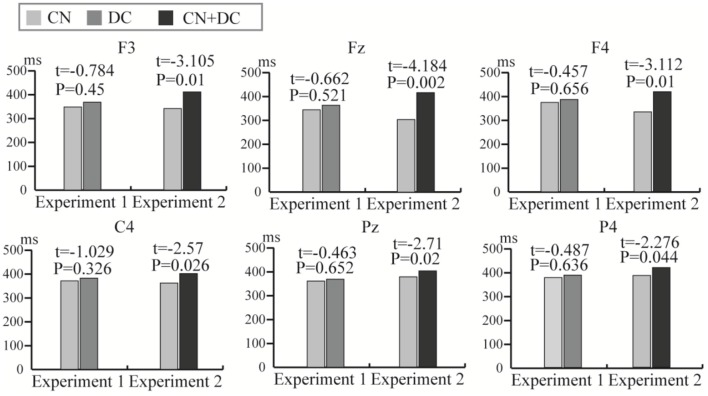
The comparison of average latency of the P300 potential between the CN and DC tasks of Experiment 1 and between the CN and CN + DC tasks of Experiment 2 at the F3, Fz, F4, C4, Pz, and P4 electrodes. CN: task involving counting the number of intensified targets, DC: task involving distinguishing targets’ color, CN + DC: task involving simultaneously counting the number of intensified targets and distinguishing their color.

We computed the latency of the P300 potential in the DC and CN tasks of Experiment 1 and the CN + DC and CN tasks of Experiment 2. We then conducted a pairwise *t*-test for the latency between the DC and CN tasks, as well as that between the CN + DC and CN tasks. The results of the statistical analysis showed that although the average latencies of the P300 potential induced in the DC and CN + DC tasks were both longer than those elicited in the CN task; there was no significant difference in the latency of the P300 potential between the DC and CN tasks in Experiment 1, while the latency of the P300 potential between the CN + DC and CN tasks at the F3, Fz, F4, C4, Pz, and P4 electrodes showed a significant difference in Experiment 2 (Figure 10).

### Offline Classification Results

**Figure 11:**
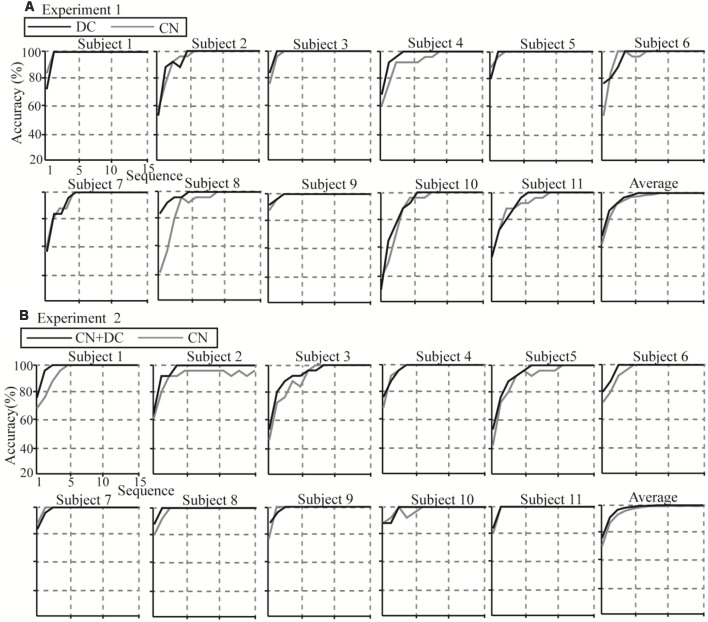
Individual and average accuracies of the P300-speller for all subjects in the two experiments. **(A)** Individual and average accuracies of the DC and CN tasks for the 11 subjects in Experiment 1. **(B)** Individual and average accuracies of the CN + DC and CN tasks for the 11 subjects in Experiment 2. CN: task involving counting the number of intensified targets, DC: task involving distinguishing targets’ color, CN + DC: task involving simultaneously counting the number of intensified targets and distinguishing their color.

Individual and average accuracies in the DC/CN + DC and CN tasks for the 11 subjects in Experiments 1 and 2, with different superposition times, are shown in Figure 11. Using more superposition times can improve the robustness of the spelling process, but leads to lower ITR. Thus, it is important to improve the performance of the P300-speller so as to achieve 100% accuracy with fewer superposition times. From the average accuracies in Experiments 1 and 2, we observed that the superposition times in the DC and CN + DC tasks were both less than the superposition times in the CN task when the average accuracies reached 100% (six, five, and four times in the CN, DC, and CN + DC tasks, respectively). Further, the average accuracies in the DC and CN + DC tasks were both higher than those in the CN task at each superposition time before the accuracy reached 100%. We conducted a *t*-test for the accuracies at each superposition time of the DC/CN + DC and CN tasks in Experiments 1 and 2 before the accuracy rates reached 100%.

We found that although the average accuracies in the DC task were greater than those in the CN task before the accuracies reached 100%, there was no significant difference between the two (Table 1). In Experiment 2, we found significant differences between the CN and CN + DC tasks for one, two, three, and four superposition times (Table 1).

**Table 1:** Results of paired *t*-tests between the CN and DC tasks, and between the CN and CN + DC tasks for one, two, three, and four superposition times.

Superposition times	Tasks	*t*	*P*
One	(CN, DC)	−1.203	0.257
	**(CN, CN + DC)**	**−4.224**	**0.002**
Two	(CN, DC)	−1.505	0.163
	(CN, CN + DC)	**−2.232**	**0.050**
Three	(CN, DC)	−0.167	0.875
	**(CN, CN + DC)**	**−2.319**	**0.043**
Four	(CN, DC)	**−1.936**	**0.062**
	**(CN, CN + DC)**	**−2.206**	**0.052**

*CN: task involving counting the number of intensified targets; DC: task involving distinguishing targets’ color; CN + DC: task involving simultaneously counting the number of intensified targets and distinguishing their color*. The significance of bold values is *p* < 0.05.

### Online Experiment Results

We recorded the online accuracies and ITRs of 10 subjects for the three types of tasks. The results are shown in Table 2.

**Table 2:** Online classification accuracy and ITR of each subject for the three tasks.

	Accuracy (%)			ITR (bit/min)		
	CN	DC	CN + DC	CN	DC	CN + DC
Subject 1	70	80	73	15.0	18.7	16.1
Subject 2	63	73	70	12.7	16.1	15.0
Subject 3	97	100	97	26.3	27.5	26.3
Subject 4	83	87	90	19.7	21.5	22.9
Subject 5	77	80	87	17.5	18.7	21.5
Subject 6	87	80	93	21.5	18.7	24.2
Subject 7	77	77	83	17.5	17.5	19.9
Subject 8	83	90	90	21.5	22.8	22.8
Subject 9	80	80	83	18.7	18.7	21.5
Subject 10	60	67	67	11.7	14.0	14.0
Avg. ± SD	77.7 ± 11.12	81.4 ± 9.17	83.3 ± 10.19	18.21 ± 4.39	19.42 ± 3.76	20.42 ± 4.12

*ITR: information transfer rate, CN: task involving counting the number of intensified targets, DC: task involving distinguishing targets’ color, CN + DC: task involving simultaneously counting the number of intensified targets and distinguishing their color, SD: standard deviation*.

The average online accuracies and ITRs in the DC and CN + DC tasks were both higher than the average online accuracies in the CN task, and the average online accuracy and ITR in the CN + DC task was higher than those in the DC task. The results of the paired *t*-tests for each pair of tasks show that the accuracy and ITR were significantly different between the CN and CN + DC tasks (accuracy: *t* = −6.244, *P* < 0.001; ITR: *t* = −6.111, *P* < 0.001), while there was no significant difference in the accuracies and ITRs between the CN and DC tasks, or between the DC and CN + DC tasks [(CN, DC): accuracy, *t* = −2.247, *P* = 0.051, ITR, *t* = −2.044, *P* = 0.071; (DC, CN + DC): accuracy, *t* = −1.036, *P* = 0.327, ITR, *t* = −1.302, *P* = 0.225, respectively].

## Discussion

The purpose of this study was to verify the effect of DC-based active mental and CN + DC tasks on the performance of the P300-speller. We found that the amplitudes of the P300 potential induced in the DC and CN + DC tasks were larger than those induced in the CN task, and the largest amplitudes were induced in the CN + DC task. The offline and online results further showed that the average accuracies in the DC and CN + DC tasks were greater than those in the CN task.

### ERP and Latency Analyses

The ERP analysis revealed that the stimuli in the DC and CN + DC tasks all elicited amplitudes and latency different from those elicited in the CN task in the two experiments.

**Figure 12:**
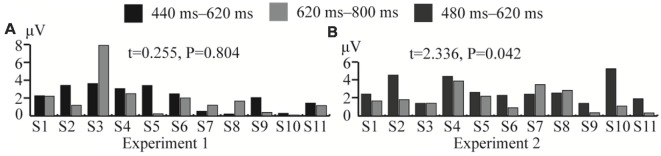
Comparison of average amplitude difference between different time windows in Experiments 1 and 2 at the Pz electrode. **(A)** Comparison of average amplitude difference of (DC-CN) between 440–620 and 620–800 ms for 11 subjects in Experiment 1. **(B)** Comparison of average amplitude difference of (CN + DC)-CN between 480–620 and 620–800 ms for 11 subjects in Experiment 2. CN: task involving counting the number of intensified targets, DC: task involving distinguishing targets’ color, CN + DC: task involving simultaneously counting the number of intensified targets and distinguishing their color (CN + DC)-CN: the waveform generated by subtracting waveform in CN task from that in CN + DC task. DC-CN: the waveform generated by subtracting waveform in CN task from that in DC task.

In Experiment 1, the significant difference between the ERP waveforms elicited in the DC and CN tasks was mainly localized at the parietal-central area between 440 and 800 ms after stimulation (Figure 6); the ERP waveform induced between 440 and 620 ms may correspond to P3b (Figure 5), while that found between 480 and 800 ms may be the LPC (Figure 5). Polich (2007) proposed a decomposition of the P300 potential into P3a and P3b subcomponents. The latter is a neural marker of cognitive processing and decision-making operations that has a more parietal distribution and longer latency, usually between 280 and 600 ms (Polich, 2007). The LPC is an ERP component related to selection response in memory-updating operations and is a positive deflection during 400–800 ms at the central-parietal area (Guo et al., 2010). The LPC and P3b have similar latencies and scalp-topography distribution, but the former could reflect even later mental processes than the P3b (Sellers and Donchin, 2006; Krusienski et al., 2008). Therefore, the ERP waveform with a significant difference at the parietal area between 440 and 620 ms may indicate an overlap of the LPC with the P3b; however, we could not determine whether the increased amplitude between 440 and 620 ms in the DC task corresponds to the increased LPC amplitude or an increase in both P3b and LPC amplitudes. Therefore, we compared the amplitude increase in the two different time windows (440–620 and 620–800 ms) by averaging the difference in the waveform observed in the (DC-CN) during the two time windows (Figure 12A) at the Pz electrode. The result showed that there was no significant difference in the amplitude increase between 440 and 620 ms and between 620 and 800 ms. We speculated that the increased amplitude during the earlier time window in the DC task may correspond to an increased LPC amplitude. Some studies have proposed that the task of discriminating the stimuli’s features provides a more active selection response than the counting task, and the results from these studies showed that the LPC amplitude increased in a more active mental task (Guo et al., 2010; Xu H. et al., 2013). As shown in the study by Huang and Pashler (2007), focusing on the visual features of the target can induce more obvious amplitudes. Therefore, the amplitude of LPC waveforms was higher in the DC task than that in the CN task, which may be due to the DC task eliciting a more active response than the CN task. In the study by Xu H. et al. (2013), the stimuli elicited significant ERP amplitude by changing the counting task to one involving discrimination of the auditory stimulus properties (laterality, sex to which of the sound belongs).

In Experiment 2, the significant difference between the ERP waveforms elicited in the CN + DC and CN tasks were found in two regions: the left- and right-frontal area between 320 and 480 ms (Figure 8A) and the parietal-central area between 480 and 800 ms (Figure 8B). The significant differences found in the first region may correspond to the subcomponent P3a. This subcomponent occurs after a novel event and features more frontal distribution; its latency is usually between 220 and 400 ms, which is associated with attention processing (Polich, 2007). Corbetta et al. (1991) proved that focusing on multiple features of a stimulus requires more attention than focusing on only one of its features. The CN + DC task was designed to require the subject to pay attention to both the number of times a target was presented and the its color, which requires more attention than the CN task. Riccio et al. (2013) investigated the influence of attention on the performance of a P300-based BCI system and found a significant positive correlation between attention and the P300 amplitude. Thus, a significant increase in the P3a amplitude may be due to increased attention. Similarly, as the ERP waveform with a significant difference in the parietal area between 480 and 620 ms indicated a possible overlap of the LPC with the P3b (Figures 7, 8), we compared the amplitude increase in two different time windows (480–620 and 620–800 ms) by averaging the difference of [(CN + DC)-CN] across the two time windows (Figure 12B). We found a significant difference in the amplitude increase between 480 and 620 and that between 620 and 800 ms (*P* < 0.05). This finding supported our speculation that the increased amplitude between 480 and 620 ms in the CN + DC task corresponded to both P3b and LPC amplitudes. In a study on the assessment of mental workload, the P3b amplitude was found to increase when the subjects’ task was changed from a counting-number to a reverse counting-number type because the reverse counting number task increased the difficulty of the decision-making operation (Horat et al., 2016). In the present study, the subjects were required to count the number of targets and distinguish their colors at the same time in the CN + DC task, which doubled the decision-making operation and increased the difficulty of the CN task. Thus, the P3b amplitude was heightened in the CN + DC task relative to that in the CN task. Furthermore, the subjects were required to engage in a more complex selection response (the number of times a target was presented and its color) in the CN + DC task than that in the CN task, as the former presented a more active mental task to the participants; the significant increase in the LPC amplitude in the CN + DC task is thus accounted for.

In addition, we found that the amplitude difference between the CN + DC and CN tasks was larger than that between the DC and CN tasks. We subtracted the waveform of the CN task from that of the CN + DC task and the waveform of the CN task from that of the DC task and compared the two results. We found that the two subtracted waveforms have significant differences at the left-frontal, right-frontal, and parietal areas (Figure 9). This indicated that the CN + DC task required more attention, cognitive processing, and decision-making processes than not only the CN task but also the DC task. This finding validated the settings of our experimental tasks, which were designed to make the CN + DC task more difficult.

In addition to inducing significantly different ERP amplitudes in the DC and CN + DC tasks compared with the CN task; the latency of ERP was also different between the DC and CN tasks, and between the CN + DC and CN tasks. In Experiments 1 and 2, the average latency of ERP waveforms in the DC and CN + DC tasks were both longer than that in the CN task (Figure 10). In Experiment 1, however, there was no significant difference in latency; the significant differences were localized to the frontal and right-parietal areas in Experiment 2 (Figure 10). Some studies demonstrated that the more difficult the task, the longer is the latency (Bennington and Polich, 1999; Katayama and Polich, 1999). In this context, the difficulty of the DC task was not obviously different from that of the CN task, while the difficulty of the CN + DC task relative to the CN task was clear. This is consistent with our having designed the active mental tasks according to difficulty.

### Classification Accuracies and ITRs

From the offline and online results, the superposition times (before the accuracy reached 100%) of the offline experiment in the DC and CN + DC tasks were both less than that in the CN task; the ITRs of the online experiment in the DC and CN + DC tasks were both larger than that in the CN task. The average offline and online accuracies in the DC and CN + DC tasks were both larger than that in the CN task. This shows that the performances of the P300-speller based on the DC and CN + DC tasks were better than that based on the CN task. We found a significant difference between the CN + DC and CN tasks in offline accuracies, online accuracies, and ITRs (Figure 11; Tables s 1, 2), which is consistent with the ERP results: the larger amplitude and longer latency of the CN + DC task. Xu H. et al. (2013) found that the benefit of the active mental task paradigm likely originates from the P300 latency and amplitude. Therefore, the significant improvement in the performance of the P300-speller based on the CN + DC task is likely to be related to the higher amplitude and longer latency than that in the performance of the P300-speller based on the CN task. There was no significant difference between the offline and online results of the DC and CN tasks, but our results indicated that although there was no significant improvement in the performance of the P300-speller based on the DC task compared with that based on the CN task, there was a trend of improvement (offline accuracies of three superposition times (CN, DC): *t* = −1.936, *P* = 0.062; accuracy of online (CN, DC): *t* = −2.247, *P* = 0.051; ITR of online (CN, DC): *t* = −2.044, *P* = 0.071). Further, the DC task is meaningful for further improving the performance of P300-speller, which provides certain references for us to change the task from the perspective of visual features. For example, when the stimuli are of different shapes, such as triangles, rectangles, etc., we can ask the subjects to discriminate among different shapes instead of counting.

## Conclusion

This study investigated the influence of different active mental tasks on the performance optimization of the P300-speller. We found that mental tasks based on color distinction and varying in difficulty induce different ERP amplitudes that are more obvious than those elicited by a counting task. Active mental tasks based on task difficulty can, therefore, significantly improve the accuracy and ITR of the P300-speller and optimize its performance.

## Ethics Statement

This study was carried out in accordance with the recommendations of “the ethics committee of Changchun University of Science and Technology (CUST)” with written informed consent from all subjects. All subjects gave written informed consent in accordance with the Declaration of Helsinki. The protocol was approved by the “the ethics committee of Changchun University of Science and Technology (CUST)”.

## Author Contributions

The manuscript was written with contributions from all authors. All authors have approved the final version of the manuscript. QL designed experiments. ZL performed experiments. ZL and JY analyzed experimental results. NG checked and verified the experimental results. QL and ZL wrote the manuscript.

## Conflict of Interest Statement

The authors declare that the research was conducted in the absence of any commercial or financial relationships that could be construed as a potential conflict of interest.
